# Association between vitamin D concentrations and knee pain in patients with osteoarthritis

**DOI:** 10.7717/peerj.4670

**Published:** 2018-04-24

**Authors:** Murat Cakar, Semih Ayanoglu, Haluk Cabuk, Metin Seyran, Suleyman Semih Dedeoglu, Hakan Gurbuz

**Affiliations:** 1 Department of Orthopaedics and Traumatology, Okmeydanı Training and Research Hospital, Istanbul, Turkey; 2 Department of Orthopaedics and Traumatology, Medipol Universty Hospital, Istanbul, Turkey

**Keywords:** Vitamin D, Osteoarthritis, Pain, Knee

## Abstract

**Objectives:**

Osteoarthritis (OA) and vitamin D deficiency are common health conditions in older people. Whether vitamin D concentration is associated with knee OA is controversial. In this study, we aimed to determine the association between serum concentrations of vitamin D and osteoarthritic knee pain.

**Subjects and Methods:**

Vitamin D concentrations were measured with the 25 hydroxyvitamin D test in patients presenting with clinical symptoms of primary knee osteoarthritis. Osteoarthritis was graded on the Kellgren-Lawrence grading scale from anteroposterior and lateral radiographs. Height, weight, and body mass index (BMI) were recorded. Patients completed a 10-cm visual analogue scale (VAS) for indicating pain and the Western Ontario and McMaster Universities Arthritis Index (WOMAC). Vitamin D concentration was defined as severely deficient (<10 ng/mL), insufficient (10 to 19 ng/mL), or normal (20 to 50 ng/mL).

**Results:**

Of 149 patients (133 women), the mean age was 63.6 years. Mean vitamin D concentration was 11.53 ng/mL, and 90% patients were vitamin D deficient. Mean WOMAC score was 57.2, and VAS pain score was 7.5. Kellgren-Lawrence grade was 2 for 10 patients, grade 3 for 61, and grade 4 for 88. Mean BMI was 33.4. Mean values of VAS, WOMAC, and BMI did not differ by vitamin D status.

**Conclusion:**

Serum vitamin D concentration is not associated with knee pain in patients with osteoarthritis.

## Introduction

The incidence of vitamin D deficiency is 25% in the general population and more than 40% in the elderly population in US and it is a common health problem (Heath & Elovic, 2006). Vitamin D is important to normal bone and cartilage metabolism. Insufficient concentrations of vitamin D adversely effect calcium metabolism, osteoblastic activity, matrix ossification, bone density, and articular cartilage turnover (Gröber et al., 2013; Corvol et al., 1981; Cranney et al., 2008; Norman, Roth & Orci, 1982; Tetlow & Woolley, 2001; Heath & Elovic, 2006). Vitamin D can reduce bone turnover and cartilage degradation, thus potentially preventing the development and progression of knee osteoarthritis (Holick, 2006; McAlindon et al., 2013). Epidemiological studies showed that low serum 25-hydroxyvitamin D levels were associated with greater knee pain a higher prevalence of radiographic knee osteoarthritis and higher risk of progression (McAlindon et al., 1996; Bergink et al., 2009). It has also anti-inflammatory properties, proinflammatory cytokine production in states of vitamin D deficiency might alter central pain processing, thereby increasing mechanical pain sensivity (Müller et al., 1992). A cross-sectional study reported that the prevalence of vitamin D deficiency was 72% in patients with widespread musculoskeletal pain (Çidem et al., 2013).

Osteoarthritis (OA) is a degenerative joint disease involving the cartilage and many of its surrounding tissues. In addition to damage and loss of articular cartilage, there is remodelling of subarticular bone, osteophyte formation, ligamentous laxity, weakening of periarticular muscles, and, in some cases, synovial inflammation. Pain is the major clinical symptom in knee OA joint.

Osteoarthritis (OA) and vitamin D deficiency are common health conditions in older people. Whether vitamin D concentration is associated with knee OA is controversial. Vitamin D concentrations have been associated with worsening OA, increase in cartilage loss, development of knee osteophytes, worse radiological grading, and poorer functional status. It has been hypothesized that vitamin D supplementation might be a feasible and cost effective strategy for controlling symptoms and inducing structural improvement in patients with knee OA. However, results for the correlation between low blood concentrations of vitamin D and knee OA are conflicting.

We aim to determine whether there is a correlation between knee pain and serum vitamin D levels in patients with knee OA.

## Subjects and Methods

The study was approved by the institutional review board of Okmeydanı Training and Research Hospital (approval number: 486700710). All patients provided written informed consent before being enrolled in the study.

In this cross-sectional study, all patients admitted to Orthopaedics and Traumatology Clinic with clinical and radiological evidence of knee OA between 1 June and 31 August 2013, were eligible for enrollment. We excluded patients with underlying endocrine pathology, inflammatory diseases, patients who take medications affecting D vitamin status or a history of knee trauma and surgery.

Height and weight were measured in examination room by the same surgeon. Body mass index (BMI; weight (kg)/height (m^2^)) was calculated. Anteroposterior and weight-bearing radiographs of the knee were taken with the same radiographic equipment. The severity of OA was determined by the Kellgren-Lawrence system from radiographs. Grades 1 and 2 were considered to be mild and grades 3 and 4 as severe.

Knee pain was assessed by a self-administered questionnaire that include a 10-cm visual analogue scale (VAS) and the Western Ontario and McMaster Universities Arthritis Index (WOMAC). The WOMAC consists of 24 items, each scored from zero to 4, yielding a total score from zero to 96. Higher scores indicate more severe disease. There are five items for pain, two for stiffness, and 17 for functional limitations, such as stair use, walking, getting in or out of a bath, and household duties.

Fasting morning blood samples were obtained from all patients and were anti-coagulated with EDTA. The plasma was separated by centrifugation. Plasma 25-OH D concentration was determined by radioimmunoassay kits via elisa (Anti 25(OH) Vitamin D monoclonal, Beckman Coulter, Oslo, Norway). Vitamin D concentration was defined as severe deficient (<10 ng/mL), insufficient (10 to 19 ng/mL), or normal (20 to 50 ng/mL).

## Statistical Methods

Frequencies and percentages were compared among the three vitamin D status groups with Chi-square tests. Correlations between vitamin D concentrations and WOMAC, VAS pain scores, and BMI were assessed with multiple stepwise regression analysis. Alpha was set at 0.05. Data were analyzed with the Statistical Package for Social Sciences (SPSS) version 17.5. All data conformed to the assumptions of the test used to analyze them.

## Results

Of the 142 enrolled patients (126 woman), mean age was 64 years. Vitamin D status was deficient in 77, insufficient in 51, and normal in 14. Kellgren-Lawrence grade was 2 in 10 patients, 3 in 57 patients, and 4 in 85. Demographic data was shown on Table 1.

**Table 1:** Clinical characteristics of 149 patients with osteoarthritis of the knee in a study of the association between vitamin D concentrations and knee pain.

Characteristic	Mean (SD)	Median	Minimum	Maximum
Vitamin D concentration, ng/mL	11.5 (9.0)	9.3	2.4	63
^*^WOMAC score	57.2 (17.6)	60	10	86
Pain score	7.5 (1.9)	8	1	10
Height, cm	159.0 (7.5)	159	138	186
Weight, kg	84.2 (11.7)	82	59	120
Body mass index	33.4 (4.5)	33.3	23.4	49.9

**Notes.**

* WOMAC, Western Ontario and McMaster Universities Arthritis Index.

Aside from a borderline-significant difference in age, there were no statistically significant or clinically important differences among the three vitamin D status groups (Table 2). Age was weakly but significantly correlated with Kellgren-Lawrence grade, vitamin D concentration, WOMAC score, and BMI (Table 3). WOMAC scores were moderately and significantly correlated with pain scores and Kellgren-Lawrence grade (Table 3).

**Table 2:** Linear and multiple regression analysis of demographic variables with WOMAC scores.

	Linear regression analysis			Multiple regression analysis (Stepwise)		
	Mean	SD	*p*	Beta	*R*	*p*
Age	63.5	8.98	0.062			
K-L grade	3.45	0.62	0.026	0.302	0.302	0.000
Vit D	11.57	8.98	0.506			
BMI	33.47	4.49	0.428			

**Notes.**

Dependent variable: Womac scores.

**Table 3:** Linear and multiple regression analysis of demographic variables with VAS scores.

	Linear regression analysis			Multiple regression analysis (Stepwise)		
	Mean	SD	*p*	Beta	*R*	*p*
Age	63.5	8.98	0.781			
K-L grade	3.45	0.62	0.002	0.255	0.225	0.002
Vit D	11.57	8.98	0.870			
BMI	33.47	4.49	0.445			

**Notes.**

Dependent variable: VAS scores.

## Discussion

The most importing finding of our study was there was no correlation between knee pain and vitamin D concentrations in knee OA patients. Effect of vitamin D on knee OA and pain have controversial results in literature. Although large cohort studies demonstrated that serum vitamin D levels is an independent predictor of knee pain progression (Muraki et al., 2011), small to moderate effect on pain control revealed by Diao, Yang & Yu (2017) in patients with knee OA via vitamin D supplemantation. They also conclude that effect of vitamin D supplemantation on knee pain is no more than 10% improvement (Diao, Yang & Yu, 2017).

Vitamin D sufficiency may affect cartilage metabolism. Preliminary evidence suggests that vitamin D directly affects chondrocytes in osteoarthritic cartilage, and vitamin D receptors are found in human articular chondrocytes of osteoarthritic cartilage (Tetlow & Woolley, 2001; Boyan et al., 2001).

The association of vitamin D deficiency and musculoskeletal conditions has been well studied, especially that for musculoskeletal pain, low back pain, fibromyalgia, and knee and hip OA. However, results for the correlation between low blood concentrations of vitamin D and knee OA are conflicting. Heidari, Heidari & Hajian-Tilaki (2011) reported a high prevalence of vitamin D deficiency and a significant positive association between this deficiency and knee OA in patients less than 60 years of age. Ding et al. (2009) determined from radiographs and magnetic resonance imaging that vitamin D concentrations were associated with knee pain and decreased knee cartilage loss. So vitamin D supplementation could be used for knee OA pain. Vitamin D supplementation for six months reduced oxidative protein damage, decreased pain (VAS), improved quality of life, and improved grip strength and physical performance in osteoarthritis patients, and improves lower extremity functions (Manoy et al., 2017; Shea et al., 2017). However Sanghi et al. (2013) assert a reason for the positive effect of vitamin D, which in many studies only OA patients with insufficiency vitamin D, the group most likely benefit from supplementation.

In the Framingham OA cohort study, McAlindon et al. (1996) reported that the risk of radiographic progression of knee OA over 8 years in older patients was three times as high in the middle and lowest tertiles for baseline serum concentrations of vitamin D as it was in the highest tertile. Bergink et al. (2009) also reported that vitamin D concentrations were associated with an increased risk for progression of OA. However Konstari et al. (2012) reported no significant association between vitamin D concentrations and the risks of incident knee or hip OA. Felson et al. (2007) also found that vitamin D concentrations were not associated with radiographic worsening and cartilage loss or the radiologic features of the OA (Al-Jarallah et al., 2012).

Pain is a common presenting symptom in patients with OA of the knee. However, why some people with knee OA report greater pain intensity than others, even when radiographic evidence of disease severity is comparable, remains unclear. Pain sensitization, is an important concept to explain this difference. Sensitization of the peripheral and central pathways that process nociceptive information is also an important contributor to the clinical presentation of pain in knee OA (Fingleton et al., 2015). Pain suppression by the central nervous system including top down inhibition of musculoskeletal pain might also become dysfunctional if vitamin D is low (Roesel, 2009). A study on rodents in which a vitamin D deficient diet caused mechanical muscle hypersensitivity together with increase nociceptive skeletal muscle innervation, even before muscle or bone pathology occurred (Tague et al., 2011). In our study we found no correlation between knee pain and vitamin D deficiency, but all of our patients were Kellgren-Lawrence grade 2 or more which could be a late phase in learned pain and pain sensitization. This does not mean than low levels of vitamin D play a critical role in OA pathogenesis and progression. Straube et al. suggested that the evidence for treating chronic painful conditions with vitamin D supplementation in adults is poor (Straube et al., 2015), although others have reported that moderate vitamin D deficiency independently predicts incident, or worsening of knee pain over 5 years (Laslett et al., 2014). Muraki et al. (2011) reported that lower tertiles of vitamin D concentrations might be associated with increased knee pain rather than with radiographic changes.

We found no study reporting that serum vitamin D concentration predicted pain in knee OA. Studies that did evaluate vitamin D and pain looked at changes in pain over time.

### Limitations of the Study

The number of patients was small. Due to natural selection of knee OA patients, the majority of our patients were female and overweight. We did not use magnetic resonance imaging, which could have provided us with more information about early knee OA. There could be many factors which could affect the perception of pain so we could not be sure that the pain scores were directly due to knee pain. A control group with knee OA that does not have knee pain could be more appropriate for a comparison. Also, due to the cross sectional design of the study, we did not follow up on the patients’ knee OA progressions and vitamin D levels.

## Conclusions

We found no evidence that vitamin D concentrations measured at the first visit of patients with OA of the knee were associated with knee pain based on our data. Low levels of vitamin D may not play a critical role in OA pathogenesis and progression. More studies are needed for vitamin D role in OA pathophysiology and pain perceptions.

## Additional Information and Declarations

### Data Availability

The following information was supplied regarding data availability: The raw data are provided in the tables.

## References

[ref-1] Al-Jarallah KF, Shehab D, Al-Awadhi A, Nahar I, Haider MZ, Moussa MA (2012). Are 25(OH)D levels related to the severity of knee osteoarthritis and function?. Medical Principles and Practice.

[ref-2] Bergink AP, Uitterlinden AG, Van Leeuwen JP, Buurman CJ, Hofman A, Verhaar JA, Pols HA (2009). Vitamin D status, bone mineral density, and the development of radiographic osteoarthritis of the knee: The Rotterdam Study. Journal of Clinical Rheumatology.

[ref-3] Boyan BD, Sylvia VL, Dean DD, Schwartz Z (2001). 24,25-(OH) (2) D (3) regulates cartilage and bone via autocrine and endocrine mechanisms. Steroids.

[ref-4] Çidem M, Kara S, Sari H, Özkaya M, Karacan I (2013). Prevalence and risk factors of vitamin D deficiency in patients with widespread musculoskeletal pain. Journal of Clinical Epidemiology.

[ref-5] Corvol M, Dumontier M, Tsagris L, Lang F, Bourguignon J (1981). Cartilage and vitamin D *in vitro*. Annales d’Endocrinologie.

[ref-6] Cranney A, Weiler HA, O’Donnell S, Puil L (2008). Summary of evidence-based review on vitamin D efficacy and safety in relation to bone health. American Journal of Clinical Nutrition.

[ref-7] Diao N, Yang B, Yu F (2017). Effect of vitamin D supplementation on knee osteoarthritis: a systematic review and meta-analysis of randomized clinical trials. Clinical Biochemistry.

[ref-8] Ding C, Cicuttini F, Parameswaran V, Burgess J, Quinn S, Jones G (2009). Serum levels of vitamin D, sunlight exposure, and knee cartilage loss in older adults: the Tasmanian older adult cohort study. Arthtitis and Rheumatism.

[ref-9] Felson DT, Niu J, Clancy M, Aliabadi P, Sack B, Guermazi A, Hunter DJ, Amin S, Rogers G, Booth SL (2007). Low levels of vitamin D and worsening of knee osteoarthritis: results of two longitudinal studies. Arthtitis and Rheumatism.

[ref-10] Fingleton C, Smart K, Moloney N, Fullen BM, Doody C (2015). Pain sensitization in people with knee osteoarthritis: a systematic review and meta-analysis. Osteoarthritis Cartilage.

[ref-11] Gröber U, Spitz J, Reichrath J, Kisters K, Holick M (2013). Vitamin D Update 2013: from rickets prophylaxis to general preventive healthcare. Dermato-endocrinology.

[ref-12] Heath KM, Elovic EP (2006). Vitamin D deficiency: implications in the rehabilitation setting. American Journal of Physical Medicine & Rehabilitation.

[ref-13] Heidari B, Heidari P, Hajian-Tilaki K (2011). Association between serum vitamin D deficiency and knee osteoarthritis. International Orthopaedics.

[ref-14] Holick MF (2006). High prevalence of vitamin D inadequacy and implications for health. Mayo Clinic Proceedings.

[ref-15] Konstari S, Paananen M, Heliövaara M, Knekt P, Marniemi J, Impivaara O, Arokoski J, Karppinen J (2012). Association of 25-hydroxyvitamin D with the incidence of knee and hip osteoarthritis: a 22-year follow-up study. Scandinavian Journal of Rheumatology.

[ref-16] Laslett LL, Quinn S, Burgess JR, Parameswaran V, Winzenberg TM, Jones G, Ding C (2014). Moderate vitamin D deficiency is associated with changes in knee and hip pain in older adults: a 5-year longitudinal study. Annals of the Rheumatic Diseases.

[ref-17] Manoy P, Yuktanandana P, Tanavalee A, Anomasiri W, Ngarmukos S, Tanpowpong T, Honsawek S (2017). Vitamin D supplementation improves quality of life and physical performance in osteoarthritis patients. Nutrients.

[ref-18] McAlindon TE, Felson DT, Zhang Y, Hannan MT, Aliabadi P, Weissman B, Rush D, Wilson PW, Jacques P (1996). Relation of dietary intake and serum levels of vitamin D to progression of osteoarthritis of the knee among participants in the Framingham Study. Annals of Internal Medicine.

[ref-19] McAlindon T, LaValley M, Schneider E, Nuite M, Lee JY, Price LL, Lo G, Dawson-Hughes B (2013). Effect of vitamin D supplementation on progression of knee pain and cartilage volume loss in patients with symptomatic osteoarthritis: a randomized controlled trial. Journal of the American Medical Association.

[ref-20] Müller K, Haahr PM, Diamant M, Rieneck K, Kharazmi A, Bendtzen K (1992). 1,25-Dihydroxyvitamin D3 inhibits cytokine production by human blood monocytes at the post-transcriptional level. Cytokine.

[ref-21] Muraki S, Dennison E, Jameson K, Boucher BJ, Akune T, Yoshimura N, Judge A, Arden NK, Javaid K, Cooper C (2011). Association of vitamin D status with knee pain and radiographic knee osteoarthritis. Osteoarthritis Cartilage.

[ref-22] Norman AW, Roth J, Orci L (1982). The vitamin D endocrine system: steroid metabolism, hormone receptors, and biological response (calcium binding proteins). Endocrine Reviews.

[ref-23] Roesel TR (2009). Does the central nervous system play a role in vitamin D deficiency-related chronic pain?. Pain.

[ref-24] Sanghi D, Mishra A, Sharma AC, Singh A, Natu SM, Agarwal S, Srivastava RN (2013). Does vitamin D improve osteoarthritis of the knee: a randomized controlled pilot trial. Clinical Orthopaedics and Related Research.

[ref-25] Shea MK, Loeser RF, McAlindon TE, Houston DK, Kritchevsky SB, Booth SL (2017). Sufficient vitamin K status combined with sufficient vitamin D status is associated with better lower extremity function: a prospective analysis of two knee osteoarthritis cohorts. Arthritis Care & Research.

[ref-26] Straube S, Derry S, Straube C, Moore RA (2015). Vitamin D for the treatment of chronic painful conditions in adults. Cochrane Database of Systematic Reviews.

[ref-27] Tague SE, Clarke GL, Winter MK, McCarson KE, Wright DE, Smith PG (2011). Vitamin D deficiency promotes skeletal muscle hypersensitivity and sensory hyperinnervation. Journal of Neuroscience.

[ref-28] Tetlow LC, Woolley DE (2001). Expression of vitamin D receptors and matrix metalloproteinases in osteoarthritic cartilage and human articular chondrocytes *in vitro*. Osteoarthritis Cartilage.

